# Prediction of dengue incidents using hospitalized patients, metrological and socio-economic data in Bangladesh: A machine learning approach

**DOI:** 10.1371/journal.pone.0270933

**Published:** 2022-07-20

**Authors:** Samrat Kumar Dey, Md. Mahbubur Rahman, Arpita Howlader, Umme Raihan Siddiqi, Khandaker Mohammad Mohi Uddin, Rownak Borhan, Elias Ur Rahman

**Affiliations:** 1 School of Science and Technology (SST), Bangladesh Open University (BOU), Gazipur, Bangladesh; 2 Department of Computer Science and Engineering (CSE), Military Institute of Science and Technology (MIST), Dhaka, Bangladesh; 3 Department of Computer and Communication Engineering (CCE), Patuakhali Science and Technology University (PSTU), Dumki, Patuakhali, Bangladesh; 4 Department of Physiology, Shaheed Suhrawardy Medical College (ShSMC), Dhaka, Bangladesh; 5 Department of Computer Science and Engineering (CSE), Dhaka International University (DIU), Dhaka, Bangladesh; Jadavpur University, INDIA

## Abstract

Dengue fever is a severe disease spread by Aedes mosquito-borne dengue viruses (DENVs) in tropical areas such as Bangladesh. Since its breakout in the 1960s, dengue fever has been endemic in Bangladesh, with the highest concentration of infections in the capital, Dhaka. This study aims to develop a machine learning model that can use relevant information about the factors that cause Dengue outbreaks within a geographic region. To predict dengue cases in 11 different districts of Bangladesh, we created a DengueBD dataset and employed two machine learning algorithms, Multiple Linear Regression (MLR) and Support Vector Regression (SVR). This research also explores the correlation among environmental factors like temperature, rainfall, and humidity with the rise and decline trend of Dengue cases in different cities of Bangladesh. The entire dataset was divided into an 80:20 ratio, with 80 percent used for training and 20% used for testing. The research findings imply that, for both the MLR with 67% accuracy along with Mean Absolute Error (MAE) of 4.57 and SVR models with 75% accuracy along with Mean Absolute Error (MAE) of 4.95, the number of dengue cases reduces throughout the winter season in the country and increases mainly during the rainy season in the next ten months, from August 2021 to May 2022. Importantly, Dhaka, Bangladesh’s capital, will see the maximum number of dengue patients during this period. Overall, the results of this data-driven analysis show that machine learning algorithms have enormous potential for predicting dengue epidemics.

## Introduction

**Fig 1:**
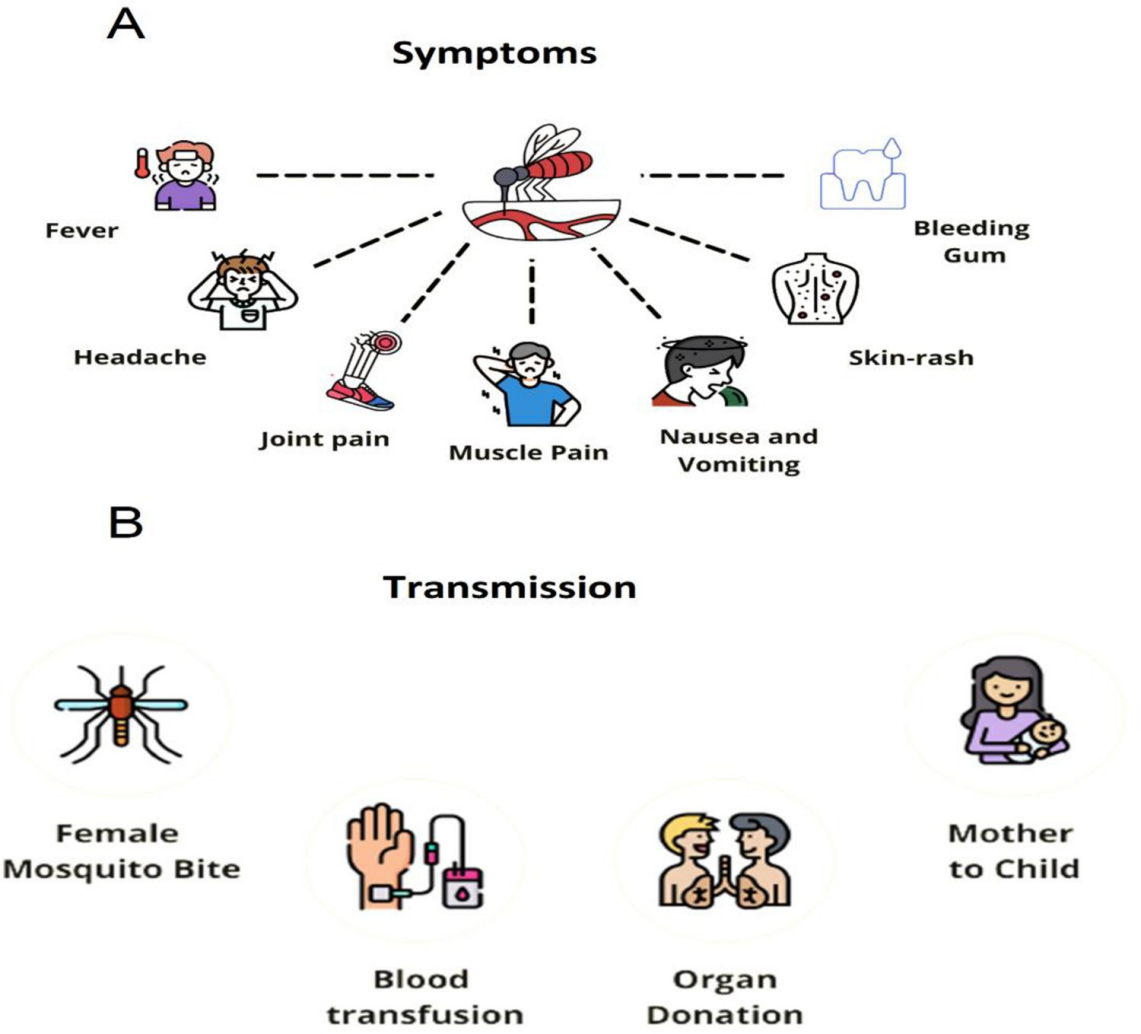
Different symptoms and transmission patterns of dengue fever.

Dengue fever is a mosquito-borne disease that is more frequent in tropical areas. Every year, between 50 and 100 million dengue patients are admitted to hospitals, and over 3 billion people reside in dengue-endemic countries [1]. **Fig 1(A)** shows the different symptoms of a dengue-affected patient caused by the dengue virus. Dengue fever is spread by a mosquito named *Aedes mosquito* at their larva stages when they carry the dengue virus. Significant symptoms do not develop early on and only become apparent when the person is in a critical condition. Seroepidemiological studies [2] demonstrate that the immune system is activated when the virus infects someone, and the body develops resistance to the homologous dengue serotype. Four different dengue virus serotypes have been identified, such as Dengue fever (DF): Aedes mosquito-borne dengue viruses (DENVs) 1–4.

After being bitten by an Aedes mosquito, a patient usually experiences a rapid rise in temperature, erythema, acute joint and nausea, muscular pain, vomiting, headache, and so on [2]. Also, there is a high probability that Dengue hemorrhagic fever (DHF) patients are more likely to develop dengue shock syndrome (DSS) [3]. 16253 dengue cases were reported within Dhaka, the capital of Bangladesh, before the Eid holiday in July during the 2019 Dengue outbreak [4]. Dengue patients were swarming into every hospital in the area at that time. All the doctors and other hospital staff members were required to work around the clock to care for the patients. After all, many people had to endure a great deal of hardship due to this tropical disease. Many people were left untreated and died due to this deadly virus. In different stages of life, the dengue virus can be found living as a parasite in the bodies of different hosts. This virus is initially found in the Aedes mosquito’s body, where it can be transmitted to humans (**Fig 1(B)**). Whenever a mosquito bites a human, the virus is transmitted through the penetration of the mosquito’s needle, which has been living in the mosquito’s body for several days. Afterward, the virus continues to replicate and multiply in the human body.

The rainy monsoon greatly benefits the Aedes mosquito’s reproduction [5]. Rain, temperature, and humidity are essential factors in the Aedes mosquito’s reproduction [6]. Bangladesh is a region where the weather is favorable for the reproduction of the Aedes mosquito during certain months, and mosquitoes of the genus Aedes can lay eggs at any time of the year [7]. Those eggs hatch when the monsoon comes or rains [8]. As a result, even a small amount of clean water can produce Aedes mosquitos, responsible for spreading the dengue virus.

This study attempts to determine the timing and place of the spreading of the dengue virus by exploring the number of dengue patients admitted to hospitals in the past years. For keeping track of the dengue patient’s data, this research followed the officially released information from the Directorate General of Health Services (DGHS), under the Ministry of Health and Family Welfare responsible for health services in Bangladesh. Along with the cases of dengue patients recorded, this research also utilized the daily weather data from the Bangladesh Meteorological Department (www.bmd.gov.bd) and an updated population census from the Bangladesh Bureau of Statistics (www.bbs.gov.bd). Out of 64 districts of Bangladesh, 11 significant districts in terms of population and geographic conditions have been chosen to predict the trend of dengue spread in this exploration. More importantly, based on records, the maximum number of dengue cases has been reported from these 11 cities (Dhaka, Faridpur, Mymensingh, Chittagong, Cox’s Bazar, Khulna, Rajshahi, Barishal, Bhola, and Sylhet). For this study, two different regression-based machine learning models (Multiple Linear Regression and Support Vector Regression) have been explored and utilized in this research, along with the developed DengueBD dataset. This article contributed to designing and developing the DengueBD dataset by predicting the spread of dengue cases in 11 different cities for the next 10 months in Bangladesh, employing two regression-based machine learning models. The performance of the two models (Multiple linear regression and Support vector regression) is evaluated using the Mean Absolute Error (MAE) on the DengueBD dataset, and Support vector regression (SVR) achieved higher accuracy in predicting dengue cases across the country.

Dengue fever is a severe disease that affects individuals across the world. As a result, several countries have been striving to solve the virus’s mystery. Researchers worldwide have been attempting to determine the virus’s origins and the optimal environment in which it can thrive.

Karim *et al*. [6] proposed a model to detect dengue utilizing multiple linear regressions. The authors utilized data from the Directorate General of Health Services (DGHS) and the Meteorological Department of Dhaka, Bangladesh, for dengue cases and climatic data from 2000 to 2008. They obtained that changes in the climate can significantly impact the spread of this virus in Dhaka city. Their study accurately forecasted a dengue breakout (≥200 cases) with the area under the ROC curve being 0.89, 95%.

Mutsuddy *et al*. [7] designed a study based on the available dengue cases that focused solely on the epidemiological elements of dengue-related morbidity and mortality in terms of climate conditions and seasonal change in Bangladesh. Their research revealed a shift in dengue incidence, with more cases reported during the pre-monsoon season due to climate change and other urban factors. However, the authors did not use any prediction approach or machine learning techniques in their investigation.

In other research, Bhatt *et al*. [9] used a combination of published literature and internet sources to create a database of 8,309 geo-located occurrence reports from 1960 to 2012. The authors created a model using the boost regression tree (BRT) framework and identified a connection between the chance of dengue incidence and inapparent using longitudinal data from 54 dengue cohort studies.

Lambrechts *et al*. [10] investigated the effects of daily temperature changes on DENV (dengue virus) transmission potential using thermodynamic models. They observed that at a mean temperature of 26°C, the percentage of infection is projected to be 99%, 88%, and 76% for (Diurnal temperature range) DTRs of 0°C, 10°C, and 20°C, respectively. The authors also stated that once mean temperatures approach 26°C, the infection risk appears to the peak.

Liu *et al*. [11] analyzed the spread out of the dengue virus from different perspectives. The authors observed that Aedes albopictus were orally infected with dengue virus 2 (DENV-2) and grown under constant temperatures (18, 23, 28, and 32° C) and a variable temperature (28–23–18°C). Their findings also revealed that, compared to the quantity of DENV-2 in salivary glands at 28°C, the amount of DENV-2 in salivary glands at 28–23–18°C was dramatically reduced.

A study has been conducted by Nagasaki University in Japan by Igarashi *et al*. [12] to observe the impact of the dengue virus on people and its control. This research includes a map of the dengue virus’s geographical spread. According to this study’s conclusions, using a net in the house can considerably prevent the transmission of this virus.

In an experiment conducted by Benelli *et al*. [13] of the University of Pisa in Italy, a novel method of mosquito control was presented, but no forecasting approaches have been designed. The authors observed that humans could prevent the spread of the virus by controlling the mosquito population and reducing the number of mosquitoes that reproduced.

Althouse *et al*. [14] compared step-down linear regression, generalized boosted regression, and negative binomial regression models to predict the incidence of dengue. For predicting a binary outcome defined by whether dengue incidence exceeded a set threshold, logistic regression, and Support Vector Machine (SVM) models were utilized. The authors obtained Dengue fever incidence data from Singapore (weekly incidence, 2004–2011) and Bangkok (monthly incidence, 2004–2011). The authors also claimed that SVM models outperformed logistic regression predicting high-incidence periods in Singapore and Bangkok. In Singapore, the Area Under the Curve (AUC) for SVM models with the 75th percentile cutoff is 0.906, whereas, in Bangkok, it is 0.960.

Uno *et al*. [15] conducted a study in the United States of America to observe the effects of the dengue virus on the human body. The life cycle of the dengue virus in the human body is depicted in this research, and the authors found that dengue vaccination can be used to reduce the number of dengue patients and the dengue vaccine, on the other hand, has been heavily criticized due to its low effectiveness in preventing the disease, at just 60%.

According to a recent editorial letter [16], dengue fever transmission season could potentially extend all year, with epidemics occurring at any time in Bangladesh. The authors calculated the change in vectorial capacity (VC) of Aedes aegypti mosquitoes at a seasonal level for all regions in Bangladesh to see how climate change affects dengue illnesses. The study’s main contribution is estimating and identifying changes in VC; however, other environmental factors such as rainfall and humidity, which could be critical drivers of dengue risk, were not considered.

Another study [17] was conducted in Bangladesh to predict dengue outbreaks between 2000 and 2008, emphasizing the impact of seasonal climate data. They created a generalized linear model based on monthly minimum temperatures, rainfall, and sunshine before the dengue season to forecast the number of yearly dengue cases. Variable selection and leave-one-out cross-validation were used to develop the best prediction model and evaluate its performance. In addition, no machine learning technique was used to anticipate the future trend of dengue incidence in Bangladesh.

Dourjoy *et al*. [18] designed a machine learning-based comparison study to predict Dengue incidences in Bangladesh using Support Vector Machine (SVM) and Random Forest (RF). The authors employed 600 patients’ survey data acquired online for their trial. According to the findings, SVM and RF produced an accuracy of 68 percent and 64 percent, respectively. However, their study concentrated solely on the pattern of the patient’s data, disregarding other factors such as metrological data, socioeconomic influence, geographic location, and weather correlation with monthly instances. Furthermore, there has been no validation of their results.

Martheswaran *et al*. [19] analyzed case report data from 2012 to 2020 to estimate dengue incidence in Singapore and Honduras using the random-sampling-based susceptible-infected-removed (SIR) model. Aside from that, the suggested model was fitted using the Bayesian Markov Chain Monte Carlo (MCMC) technique. The study’s findings suggested that their method may be used in other outbreaks, such as the ongoing COVID-19 pandemic, to comprehend the outbreak’s future dynamics better.

## Materials and methods

**Fig 2:**
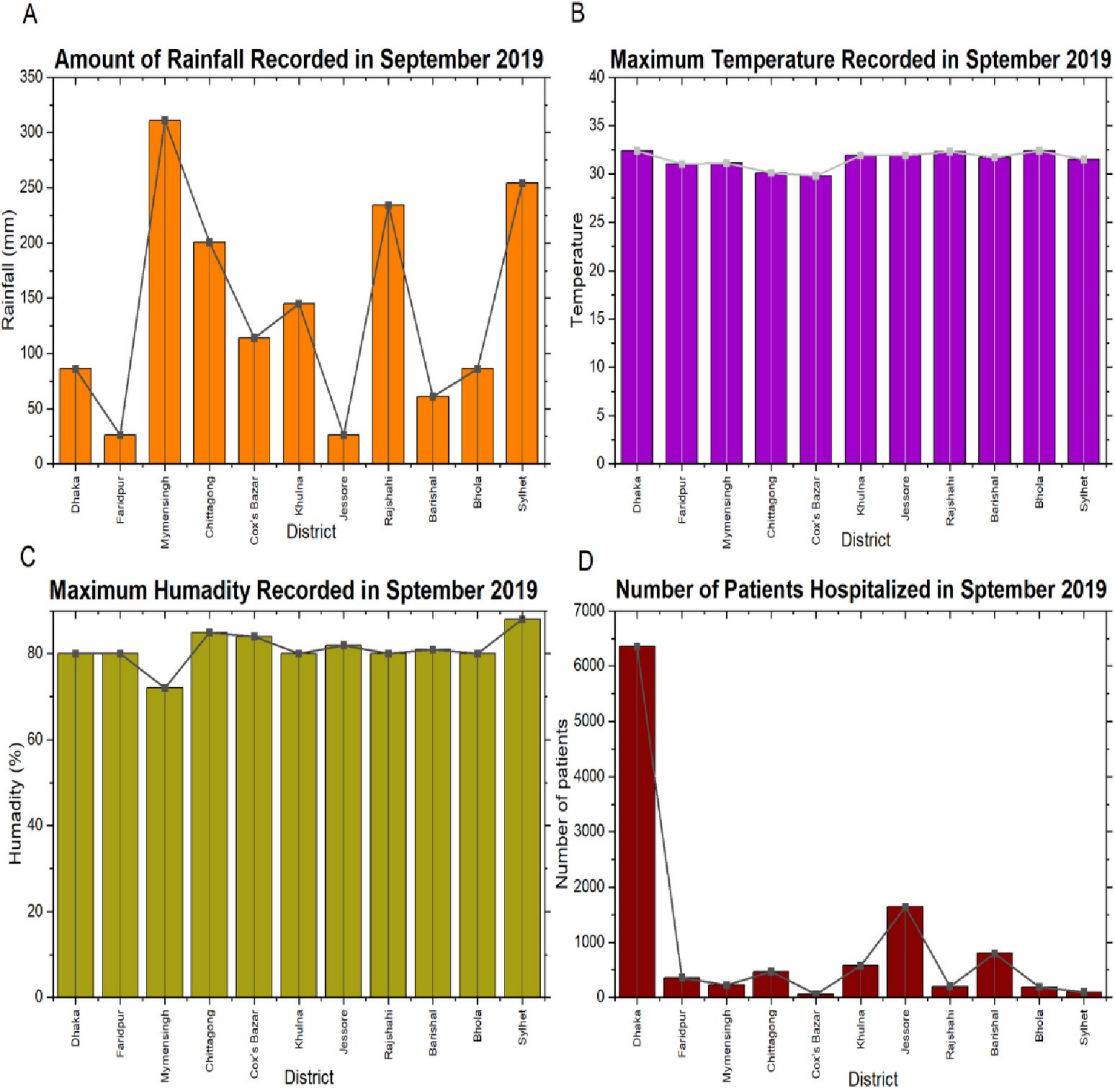
Dataset visualization for the different parameters (Rainfall, temperature, and humidity) and the number of dengue patients recorded in different districts of Bangladesh for September 2019.

A dataset named DengueBD [20] has been developed based on the available daily press released information of the Directorate General of Health Services (DGHS) [14] of Bangladesh to conduct this research. The DGHS provided daily dengue case data, while the Bangladesh Meteorological Department provided daily rainfall and temperature data from 2012 to 2019. The population consensus (2011) was collected from the Bangladesh Bureau of Statistics. During the development of the dataset, the number of patients admitted to hospitals each month in every major city in Bangladesh was included. Data were obtained from 11 of Bangladesh’s most crucial cities in terms of population and geographic location: Dhaka, Faridpur, Mymensingh, Chittagong, Cox’s Bazar, Khulna, Jessore, Rajshahi, Barishal, Bhola, and Sylhet. Apart from using the meteorological department’s data, this study also explored data from [21]. These collected data played a vital role in analyzing whether there is any correlation between the weather pattern and the number of dengue patients admitted to hospitals. **Fig 2** visualizes the amount of recorded rainfall, maximum temperature, maximum humidity, and finally, the number of hospitalized patients for September 2019. **Table 1** highlights the dataset pattern of the developed DengueBD dataset containing attributes and their description.

**Table 1:** Description of the DengueBD dataset that is created and used in this research.

DengueBD Dataset	
Attributes	Description
District	Highest affected Major cities of Bangladesh
Month	Exact month and time of admission of the patient
Rainfall	Amount of rainfall in a particular month
Maximum Temperature	The maximum temperature recorded in a particular month
Humidity	Average humidity recorded in a month
Patients	Total number of patients admitted

**Fig 3:**
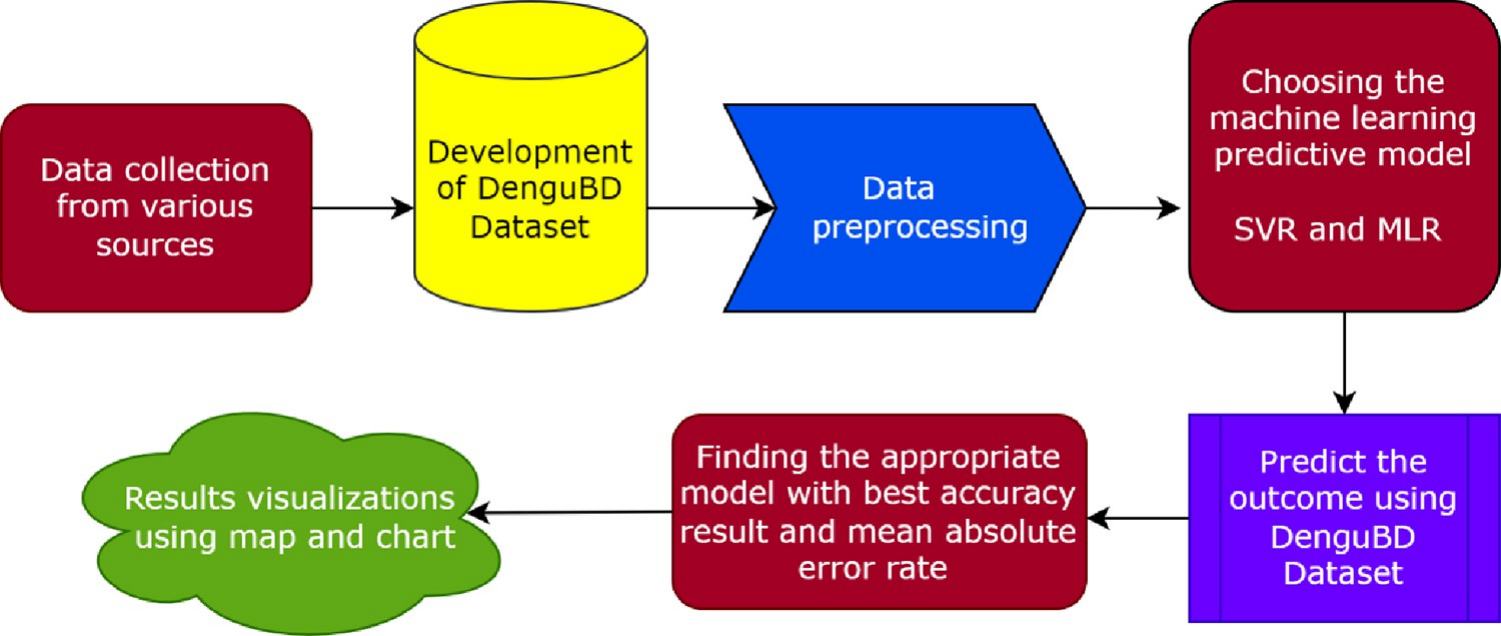
Stepwise workflow diagram for predicting the dengue cases based on the proposed algorithm.

In this section, different research methods are highlighted, along with their working procedure. **Fig 3** illustrates the workflow diagram of how this study is conducted from the initial data collection stage to prediction and visualization. Along with the data preprocessing technique, the article discusses the machine learning models that have been utilized to predict dengue cases for the upcoming years.

### Data preprocessing

DengueBD dataset is dedicatedly developed to predict the upcoming dengue cases in Bangladesh. Researchers from different domains have emphasized the importance of data preprocessing [22]. Three core python libraries have been used for preprocessing, including NumPy, Pandas, and Matplotlib. A mean calculation approach has been followed to identify and handle the missing data. For feature scaling, this research used a standardization method in which the independent variables of a dataset remain within a specific range. The equation of standardization for feature scaling is shown in (**Eq 1**), where *mean()* returns the mean of feature x, and *std()* returns the standard deviation of x.


$$\bar{X}=\frac{x-mean(x)}{std(x)}$$ (1)

Finally, for splitting the dataset into 80% training and 20% test data, we have imported the *train_test_split* method from *sklearn*.*model_selection* packages using the libraries of Scikit learn.

### Proposed prediction model

This study used two regression algorithms: Support Vector Regression (SVR) and Multiple Linear Regression (MLR).

#### Support Vector Regression (SVR)

Support Vector Regression (SVR) [23] is a machine learning algorithm proposed in this paper for predicting dengue incidents. In this regression technique, two lines operate as the decision boundary, and those decision boundaries determine a single line in the middle, referred to as a hyperplane. When applying SVR, the points within the decision boundary have been considered. A hyperplane is the best fit line that contains a maximum number of points. Consider the decision lines are located from the hyperplane at a distance *α*. Therefore, these lines can be obtained at distances ‘+*α*’ and ‘-*α*’ from the hyperplane.

Assume that the hyperplane’s equation is as follows:

$$\gamma =\omega \tilde{x}+\beta$$ (2)

The decision boundary equations then become:

$$\begin{array}{c} \omega \tilde{x}+\beta =+\alpha \\ \omega \tilde{x}+\beta =-\alpha \end{array}$$ (3)

As a result, any hyperplane that fits SVR should do so:

$$-\alpha <\gamma -\omega \tilde{x}+\beta <+\alpha$$ (4)

The primary objective of using the SVR is to determine a decision boundary that is *α* distance from the original hyperplane such that data points remain as close to the hyperplane as possible.

#### Multiple Linear Regression (MLR)

Derived from linear regression, MLR deals with time series data where it forecasts the values of one or more response variables using a set of independent variables [24]. MLR is one of the proposed and applied models to predict dengue cases in this research. The proposed prediction model has multiple independent variables that considerably correlate with the dependent variable. Therefore, this study utilized a multiple linear regression algorithm. In multiple linear regression, the model is assumed to be,

$$Y=\beta_{0}+\beta_{1}\times X_{1}+\beta_{2}\times X_{2}+\cdots {+\beta }_{m}\times X_{m}+\varepsilon$$ (5)

where *Y* indicates the target variable and *β*_0_, *β*_1_, *β*_2_,…,*β*_*m*_ are the coefficients of the model. *X*_1_, *X*_2_,…,*X*_*m*_ are the feature variables or independent variables. In multiple linear regression models, data is assumed to be normally distributed. Thus, for n observations, it holds,

$$\begin{array}{c} y_{1}=\beta_{0}+\beta_{1}\times x_{11}+\beta_{2}\times x_{12}+\ldots +\beta_{m}\times x_{1m}+\varepsilon_{1}\\ y_{2}=\beta_{0}+\beta_{1}\times x_{21}+\beta_{2}\times x_{22}+\ldots +\beta_{m}\times x_{2m}+\varepsilon_{2}\\ \vdots \\ y_{n}=\beta_{0}+\beta_{1}\times x_{n1}+\beta_{2}\times x_{n2}+\ldots +\beta_{m}\times x_{nm}+\varepsilon_{n.} \end{array}$$ (6)

Eq (6) can be written as,

$$\left(\begin{array}{c} y_{1}\\ y_{2}\\ \vdots \\ y_{n} \end{array}\right)=\left(\begin{array}{l} 1\;x_{11}\;x_{12}\;\ldots \;x_{1m}\\ 1\;x_{21}\;x_{22}\;\ldots \;x_{2m}\\ \vdots \;\vdots \;\vdots \;\vdots \\ 1\;x_{n1}\;x_{n2}\;\ldots \;x_{nm} \end{array}\right)\left(\begin{array}{c} \beta_{1}\\ \beta_{2}\\ \vdots \\ \beta_{m} \end{array}\right)+\left(\begin{array}{c} \varepsilon_{1}\\ \varepsilon_{2}\\ \vdots \\ \varepsilon_{n} \end{array}\right)$$ (7)

$$or,\;y=\hat{X}\beta +\varepsilon$$ (8)

where, in Eq (6) and Eq (7), *y* = (*y*1, *y*2…,*yn*)^*T*^ is a *n*×1 vector of *n* observations, $\hat{X}$ is the augmentation of $X=\left(\begin{array}{l} x_{11}\;x_{12}\;\ldots \;x_{1m}\\ x_{21}\;x_{22}\;\ldots \;x_{2m}\\ \vdots \;\vdots \;\vdots \\ x_{n1}\;x_{n2}\;\ldots \;x_{nm} \end{array}\right)$ which is a *n*×*m* matrix of *n* observations on each of the *m* explanatory variables,

*β* = (*β*_1_, *β*_2_,…,*β*_*m*_)^*T*^ is a *k*×1 vector of regression co-efficient and

*ε* = (*ε*_1_, *ε*_2_,…,*ε*_*n*_)^*T*^ is *n*×1 dimensional vector of random error.

In this research, the target variable is considered as Dengue suspected hospitalized patients, and the feature variables are treated as rainfall, temperature, and humidity.

## Results and discussion

This research utilized various attributes to develop a dataset, namely ***DengueBD*,** that is used to predict the number of dengue patients in the 11 different cities of Bangladesh. The experiments were conducted on a local machine using Jupyter Notebook, whose specifications are furnished in **Table 2**. Different python libraries (3.7.12) are used to experiment with NumPy (1.0.1), Pandas (1.11.0), Scikit-learn (1.0.1), and Matplotlib (3.5.1).

**Table 2:** System specifications.

System Specification for the Experiment	
RAM	8 GB
CPU	1× single core hyper threaded i.e. (1 core, 2 threads) Xeon Processors @2.3Ghz
Cache	46 MB
GPU	NVidia K80 GPU
GPU Memory	16 GB
Session Limit	9 hours
Disk Space	100GB

**Fig 4:**
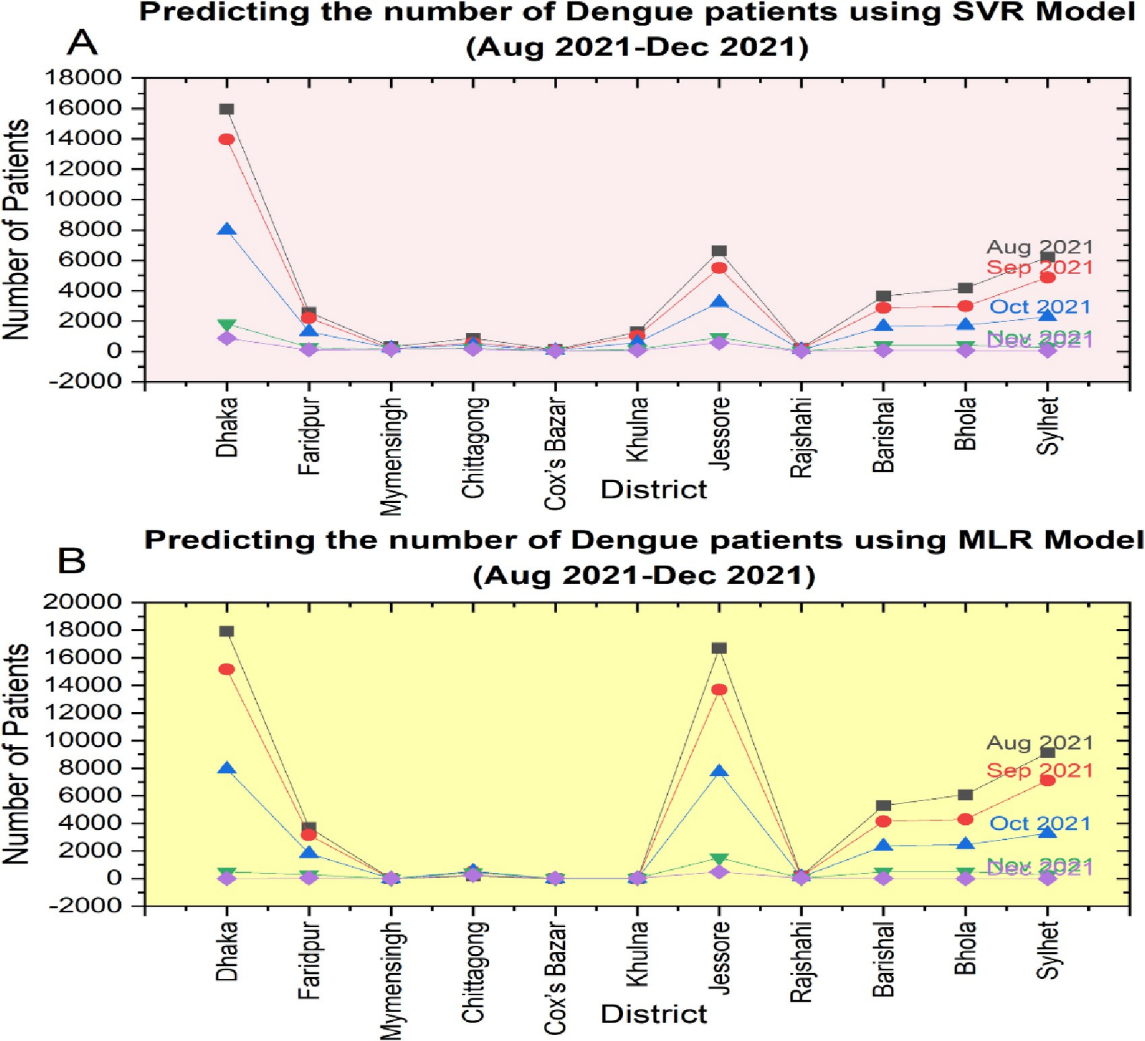
(A) shows the city-wise predicted cases of dengue viruses using the SVR Model, whereas (B) contains the MLR model results. Both models produced the outcome for the same cities of Bangladesh for the specific month of the year 2021.

Based on the experiment, we enlisted the findings in the result section. According to experiments, two sets of results have been produced, one with MLR and another with SVR. For both the regression models, this research predicted the dengue cases from August 2021 to May 2022 (10 months). MLR achieved a prediction accuracy of 67% (Mean Absolute Error (MAE):4.57), whereas SVR outperformed MLR with a prediction accuracy of 75% (MAE:4.95). In MLR, the weather is considered an independent variable, and the number of dengue patients is a dependent variable to predict future dengue patients. To better understand, **Fig 4** shows the predicted outcome for August 2021 to December 2021 for both the SVR and MLR models.

Until then, Dhaka, the capital of Bangladesh, was considered the epicenter of dengue patients, and in recent times, it experienced a significant rise in dengue cases. Support vector regression (SVR) performed well in time series prediction as a cutting-edge and powerful machine learning approach [23]. SVR was examined for tracking dengue dynamics and was compared to another regression model. An optimum cost parameter C was established to reduce overfitting and improve prediction accuracy. To choose the best SVR model, we used a cross-validation method employing Mean Absolute Error (MAE) as a performance measure. Although SVR showed good generalization performance compared to other models in this study, it can be incredibly slow in large-scale applications due to its extensive memory requirements [25]. Another key practical concern in SVR is kernel selection. The most appropriate kernel function for the dengue data should be considered when constructing the SVR model.

**Fig 4(A)** shows that the capital city was highly affected by dengue cases in August 2021. Around 7000 cases have been registered in Dhaka city for the entire month. The SVR model predicted that at the end of December 2021, dengue cases in different districts would gradually decrease. Based on **Fig 4(B)**, it can also be observed that the number of dengue patients in Dhaka city is decreasing over time according to the MLR model. With such evidence, the amount of rainfall, the highest temperature, and humidity gradually decrease every month. On the other part of the country, Jessore, another city in the southwestern region of Bangladesh, shows an upward trend and sudden spike in the number of dengue patients. A Map visualization using the MLR approach, including the observed 11 districts of Bangladesh, is shown in **Fig 5** from August 2021 to May 2022.

**Fig 5:**
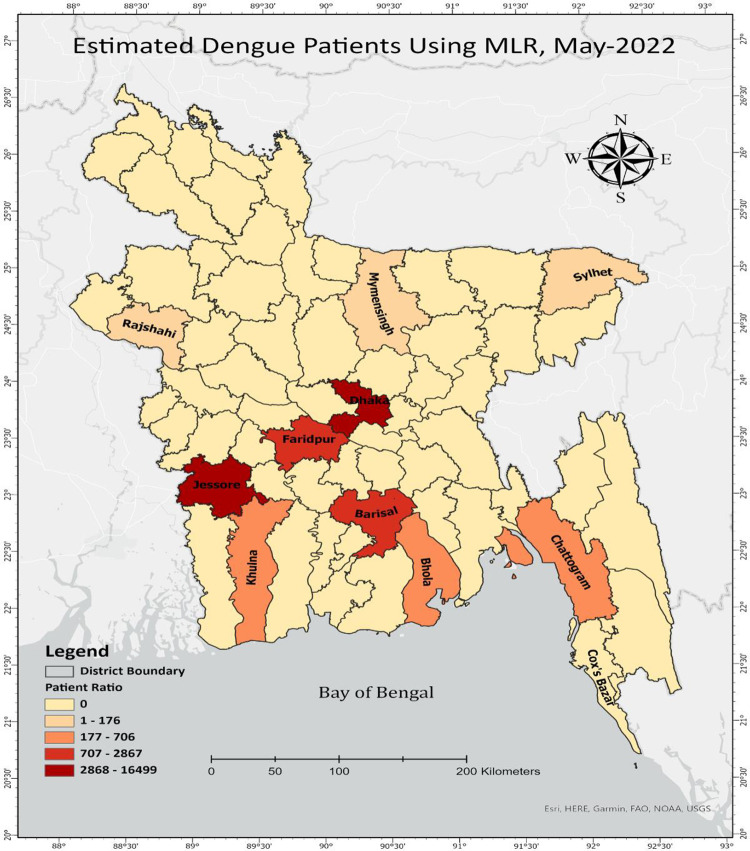
Predicting the dengue patients in 11 different cities of Bangladesh from Aug 2021 to May 2022 using Multiple Linear Regression (MLR).

**Fig 6:**
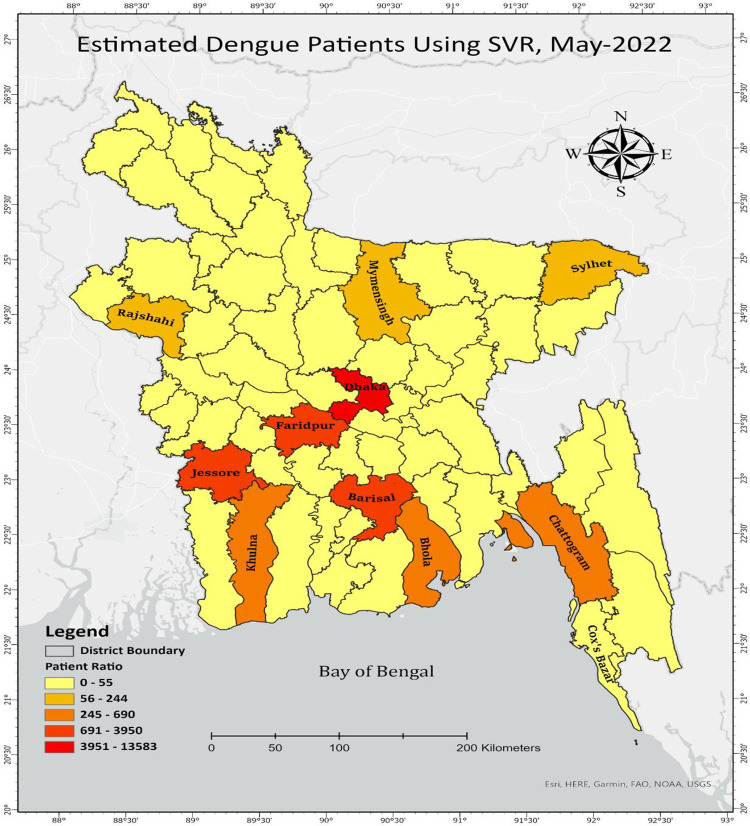
Predicting the dengue patients in 11 different cities of Bangladesh from Aug 2021 to May 2022 using Support Vector Regression (SVR).

Based on the prediction of MLR, the number of dengue patients would decrease after September 2021, but the number of dengue patients would increase again after March of the following year. **Fig 5** highlights Dhaka, Jessore, and Barishal will be the most affected district by Dengue patients until May 2022. According to the model projection, around 16500 cases may be registered in Dhaka City, followed by Jashore, Faridpur, and other cities. The Map visualization of **Fig 6** indicates that almost 13600 cases may be registered in Dhaka City, followed by Jashore, Faridpur, and other cities.

**Fig 7:**
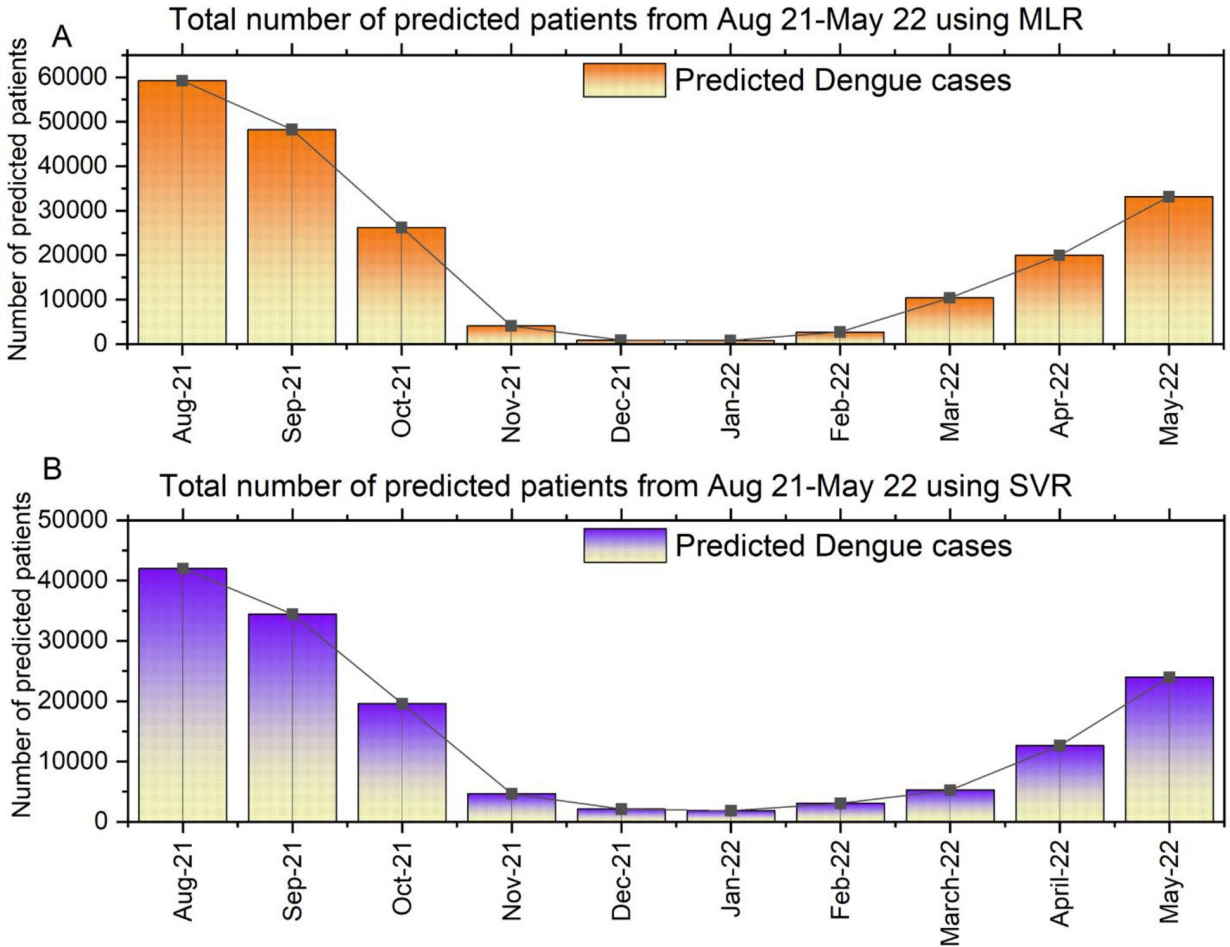
Month-wise dengue patients prediction trend using Multiple Linear Regression (MLR) and Support Vector Regression (SVR).

Interestingly the SVR prediction approach reflects that at the end of May 2022, Dhaka, the capital of Bangladesh, will be the epicenter of Dengue cases. Overall, an upcoming 10-month-based dengue patient prediction trend was also designed and highlighted in **Fig 7** for both the regression model.

This study aims to predict the number of dengue patients for the next 10 months based on the DengueBD dataset using two regression-based machine learning algorithms. Multiple Linear Regression (MLR) and Support Vector Regression (SVR) have been utilized to predict the Dengue cases in 11 different cities of Bangladesh. Apart from predicting the number of dengue patients, this research also predicted the amount of rainfall, humidity, and maximum temperature of that specific region from August 2021 to May 2022. Significant changes were observed in the predicted cases compared with MLR and SVR. Around 162272 dengue cases are predicted in 11 cities of Bangladesh for the next 10 months (Aug 2021-May 2022) using MLR. August 2021 experienced around 59244 (36%) dengue cases, which was the most compared with other months.

Interestingly, the number of cases has decreased gradually along with the changes in the environment and season in Bangladesh, and in January 2022, Bangladesh will report only 791 (0.48%) cases in the entire country, which is the lowest among all predictions. However, there will be a sudden spike in the cases during May 2022, and there is a possibility that country will observe around 33158 (20.04%) dengue cases in the upcoming year. Around 149485 people around the country will be infected by the dengue virus from Aug 2021 to May 2022, according to the prediction using SVR. It also indicated that the curve for the dengue cases will remain downward until January 2022 (1834, 1.22%), and then it experienced a quick rise ahead of February 2022. The primary reason behind the sudden increase of dengue patients in Bangladesh ahead of March is highly related to the meteorological behaviors of nature. Usually, the start of the summer season in Bangladesh is in March, and in both cases, this study overserved and predicted the upward trend in terms of dengue cases increased all over the country. Among them, based on predicted data, Dhaka, the capital city, will experience the highest number of Dengue cases in the next 10 months, and it is around 40% (65955) of total cases (162272) using MLR and 43% (65747) of total cases (149485) using SVR. Another observation this research enlisted is that dengue patients increase significantly during a specific year. As the temperature and rainfall decreased, the number of dengue patients decreased in October. This correlation indicates a strong relationship between the weather and the spread of dengue fever in the country. Managing a growing number of patients outside urban areas is challenging due to the high population density and limited capacity. In addition, many areas lack well-equipped healthcare facilities. Therefore, this study will provide a broader view for the country’s policymakers to observe and understand the future Dengue situation. Undoubtedly, the outcome of this research supports the authority to take necessary steps well ahead of time and make people aware of the situation, allowing them to protect themselves from this deadly virus. As a result, attempts to undertake risk assessments based on early warning indications must include social elements and mass movement events in addition to climate factors.

To better understand the contribution of this study, we have further designed a detailed comparison table. **Table 3** summarizes the parameters and outcomes of the previously suggested model for predicting dengue outbreaks in various countries using machine learning-based methodologies. The comparative study demonstrates that researchers from all over the country employed various data sources based on their study aims and presented projections based on their proposed model. Only studies that were dedicatedly performed on distinct ML-based or epidemic modeling-based learning algorithms were selected for the comparison.

**Table 3:** Comparative analysis of state-of-the-art research work in predicting dengue incidents globally using different data sources.

Reference	Year	Dataset source and time period	Approach	Research Methods	Performance/Findings	Study Location
Paul et al. [16]	2021	Data source: Climate data from the Bangladesh Meteorological Department. Time period: not defined	Bias-adjusted GCM data for two RCPs to estimate the VC for A. aegypti mosquitoes	Variable cost (VC) mathematical approach was used	Dengue transmission season could eventually extend to all-year-round	Bangladesh
Hossain et al. [17]	2022	Data source: Directorate General of Health Services (DGHS), Bangladesh and Bangladesh Meteorological Department (BMD) Time period: Jan 2000—Dec 2018	Prediction of dengue incidents using seasonal climate data	Quasi poisson model and corrected quasi Akaike information criteria (QAICc)	Successfully predicted the largest outbreak of dengue cases in 2018 in Bangladesh	Bangladesh
Dourjoy et al. [18]	2021	Data source: 600 dengue patients survey data and internet; 1047 data have been used Time period: not defined	Prediction of dengue fever using ML approach	SVM and Random Forest	Accuracy: 69% (SVM) and 68% (RF)	Bangladesh
Martheswaran et al. [19]	2022	Data source: Ministry of Health Infectious Disease Bulletin of Singapore, and PAHO/ WHO data of Honduras Time period: 2012–2020 (Singapore) 2015–2020 (Honduras)	Prediction of dengue fever outbreaks using climate variability and Markov chain Monte Carlo techniques	Random‑sampling‑based SIR and Bayesian Markov Chain Monte Carlo (MCMC) technique	1. Seasonal model accounted for 98.5% and 92.8% of the variance in case count in the 2020 Singapore and 2019 Honduras outbreaks 2. Climate model accounted for 75.3% and 68.3% of the variance in the Singapore and Honduras outbreaks	Singapore and Honduras
McGough et al. [26]	2021	Data source: Brazilian Ministry of Health, Brazil, and GMAO-NASA Time period: 2001–2017 (annual dengue fever cases) and 2000–2016 (daily temperature and precipitation data)	Ensemble learning approach to forecast dengue fever epidemic years in Brazil	Support Vector Machine (SVM) and ensemble learning model	Accuracy: 75% Sensitivity: 78% Specificity: 71%	Brazil
Sarma et al. [27]	2020	Data source: 209 dengue patients data collected from Dhaka and Chittagong medical college hospitals, Bangladesh Time period: not defined	ML algorithm-based dengue prediction	Decision Tree (DT) and Random Forest (RF)	Precision: 79% (DT), 67% (RF) Recall: 79% (DT), 67% (RF) F1-score: 79% (DT), 67% (RF) Accuracy: 79% (DT), 74% (RF)	Bangladesh
Salim et al. [28]	2021	Data source: private clinics, public clinics, and hospitals of Malaysia, and the source reported to the Ministry of Health through eNotifkasi, a real-time surveillance system Time period: 2013–2017	Prediction of dengue outbreak in using ML techniques	CART, SVM (linear, polynomial, rbf), Naïve Bayes, ANN	Accuracy:70%, Sensitivity: 14% Specificity:95% Precision:56% Best model: SVM (linear kernel)	Malaysia
Salami et al. [29]	2020	Data source: European Centre for Disease Prevention and Control (ECDC) and Air passenger data from the international air transport association (IATA) Time period: 2010–2015	Prediction of dengue importation using ML algorithms	Random Forest (RF), XGBoost	AUC: 94% (RF, XGBoost) Sensitivity: 79% (RF, XGBoost) Specificity:92% (RF), 93% (XGBoost)	21 European Union countries
Guo et al. [30]	2017	Data source: Guangdong provincial CDC, China National Notifiable Disease Surveillance System, China Meteorological data sharing service, Dengue search index from Baidu index website	A dengue forecast model using ML	Support vector regressor (SVR), Gradient Boosted regressor model (GBM), Negative binomial regressor (NBM), Least absolute shrinkage and selection operator (LASSO), and generalized additive model (GAM) Validation method: RMSE and R-squared measure	The SVR model accurately forecasted the 2014 large outbreak	China
Our proposed model (DengueBD dataset and multiple regression model)	2022	Data source: Directorate General of Health Services (DGHS), Bangladesh Bureau of Statistics, and Bangladesh Metrological Department Time period: Jan 2011- July 2021	Prediction of dengue incidents using the regression model	Support vector regression (SVR) and Multiple linear regression (MLR) Validation method: Mean absolute error (MAE)	Accuracy: 75% (SVR), 67% (MLR). Successfully estimated the dengue cases for the next 10 months in 11 different cities	Bangladesh

The proposed strategy is primarily based on a regression-based machine learning model that forecasts dengue outbreaks over the following ten months. However, the optimal solution cannot be guaranteed by the proposed model. As a result, the future focus of this research will be on the use of cutting-edge optimization methods on the same DengueBD Dataset. Nature-inspired optimization techniques are currently receiving greater attention. Some nature-inspired optimization algorithms that we are targeting for future research are Particle Swarm Optimization (PSO): inspired by a flock of birds or school of fish [31], and Aquila Optimizer (AO): inspired by the Aquila’s behaviors in nature during the process of catching the prey [32], Ebola Optimization Search Algorithm (EOSA): inspired by the propagation mechanism of the Ebola virus disease [33], Dwarf mongoose optimization algorithm (DMO) [34] and Reptile Search Algorithm (RSA): motivated by the hunting behavior of Crocodiles [35].

## Conclusion

Dengue epidemic containment is one of the most pressing public health issues in tropical and semi-tropical nations such as Bangladesh. After a dengue epidemic has started, countries often strive to control the mosquitoes that carry the disease. However, more sickness and death might be avoided if countries periodically monitor the circulation of viruses in mosquitos and implement control measures before massive outbreaks occur. As machine learning techniques are beneficial for classifying and forecasting dengue fever outbreaks, this study used two distinct regression-based models to predict dengue cases in 11 different districts of Bangladesh for 10 months. This study contributes to developing a comprehensive dengue dataset named DengueBD and establishes a relation between the climatic conditions with the number of dengue cases in different geographic regions of Bangladesh. Furthermore, the findings of this study will support respective concerns, authorities, the government, different healthcare organizations, and other responsible communities to understand the epidemic pattern of dengue disease in the country and act accordingly. Currently, this research lacks the availability of a standard dataset; therefore, this study cannot reflect the complete spectrum of dengue cases across the country. In the future, the prediction period can be extended by standardizing the dataset, collaborating with other organizations, and employing more Machine learning approaches to obtain a more accurate prediction.
